# Tumor-like proliferation of *CCM3* knockout endothelial cells: insights from semaxinib treatment and transcriptome profiling of co-cultures

**DOI:** 10.1186/s40478-026-02283-1

**Published:** 2026-04-20

**Authors:** Valeriia V. Saenko, Janne L. Feldmann, Dariush Skowronek, Debora Singer, Ole J. Schamuhn, Doreen Biedenweg, Sander Bekeschus, Ute Felbor, Matthias Rath, Robin A. Pilz

**Affiliations:** 1 Department of Human Genetics, Interfaculty Institute of Genetics and Functional Genomics, University Medicine Greifswald, University of Greifswald, Fleischmannstraße 43, 17475 Greifswald, Germany; 2 Department of Dermatology, Venerology, and Allergology, Rostock University Medical Center, Rostock, Germany; 3 ZIK plasmatis, Leibniz Institute for Plasma Science and Technology (INP), Greifswald, Germany; 4 Institute for Physics, University of Greifswald, Greifswald, Germany; 5 Institute for Molecular Medicine, MSH Medical School Hamburg, Hamburg, Germany; 6 Organoid Expertise Center, MSH Medical School Hamburg, Hamburg, Germany

**Keywords:** Cerebral cavernous malformations, *CCM3*, Endothelial cell, Compound screening, Semaxinib, SU5416, VEGFR2

## Abstract

Cerebral cavernous malformations (CCMs) are vascular lesions associated with severe neurological complications. Increasing evidence suggests that cancer-like mechanisms, like an abnormal expansion of *CCM3* knockout (KO) endothelial cells (ECs) in co-culture with wild-type (WT) cells, contribute to lesion formation. Yet, the underlying processes remain poorly understood. Here, we employed a human induced pluripotent stem cell (iPSC)-derived EC co-culture model to screen a cytokine inhibitor library for modulators of this tumor-like behavior. We identified the known VEGFR2 inhibitor semaxinib which selectively suppressed proliferation of WT ECs in co-culture, but not in monoculture. In contrast, *CCM3* KO cells maintained their abnormal expansion under semaxinib treatment which was unaffected by modulation of extracellular VEGFA levels. RNA-seq profiling revealed distinct transcriptional responses to semaxinib including extracellular matrix remodeling, stress signaling, and overexpression of growth factors and receptors in *CCM3* KO cells, which may contribute to their survival advantage. These findings advance our understanding of the complex interplay between WT and KO cells in CCM pathogenesis and demonstrate that the proliferative advantage of CCM3-deficient cells is not solely driven by CCM3 loss. Finally, our iPSC-based EC co-culture assay provides a scalable platform to study KO/WT interactions and may accelerate the identification of effective therapeutic strategies for CCM disease.

**Supplementary Information:**

The online version contains supplementary material available at 10.1186/s40478-026-02283-1.

## Introduction

Cerebral cavernous malformations (CCMs, OMIM: 116860, 603284, 603285) are low-flow vascular pathologies in the brain or spinal cord. They are found in approximately 0.5% of the general population and can lead to focal neurological deficits, seizures, and stroke-like symptoms due to intracerebral hemorrhage [2]. The familial form of CCM is caused by heterozygous pathogenic germline variants in *CCM1* (*KRIT1*), *CCM2* (*OSM*,* Malcavernin*), or *CCM3* (*PDCD10*) and follows an autosomal dominant inheritance. A second somatic mutation that inactivates the remaining wild-type (WT) allele in endothelial cells (ECs) is necessary for lesion development [4, 27, 32]. Despite extensive research, key aspects of CCM pathogenesis remain elusive, and no effective pharmacological treatment is available yet.

CCMs are marked by severe endothelial dysfunction, including disrupted cell-cell junctions, endothelial-to-mesenchymal transition, aberrant angiogenesis, and remodeling of the extracellular matrix (ECM) [39]. Although CCMs are not malignant tumors, recent studies have highlighted that “tumor-like” mechanisms contribute to lesion formation [4, 27, 33, 34, 38]. In particular, clonal expansion of Ccm3-deficient ECs has been demonstrated in CCM mouse models using a Confetti fluorescence reporter system [4, 20]. Similarly, abnormal expansion of human ECs following CRISPR/Cas9-mediated inactivation of the *CCM3* gene was observed in vitro, but only in direct co-culture with WT ECs [33, 36]. Furthermore, we confirmed this tumor-like behavior in co-cultures of *CCM1* or *CCM3* knockout (KO) and WT human induced pluripotent stem cell (iPSC)-derived ECs (iECs), as well as in mosaic vascular organoids [33, 38].

Our observations in co-cultures of iECs suggest that this hyperproliferation depends not only on the gene knockout, but also on a specific microenvironment [38]. This is consistent with the proposed role of non-genetic third hits in CCM formation and growth [1]. For example, immunological and inflammatory stimuli, as well as angiogenesis signaling, have been implicated as important drivers of lesion development [1, 3, 45, 49]. Targeting associated pathways might therefore improve our understanding of CCM pathogenesis and support the discovery of effective treatments for CCM. A cost- and time-efficient strategy to modulate such pathways is the screening of large compound libraries. While previous screening studies often relied on gene knockdown with RNA interference (RNAi) or animal models [11, 24, 26], our recently established 2D model of tumor-like proliferation is based on fluorescently-labeled and CRISPR/Cas9-edited iECs and enables high-throughput screening (HTS) with a simple and efficient imaging-based readout [38].

In the present work, we screened a cytokine inhibitor library in co-cultures of WT and *CCM3* KO iECs and selected the compound semaxinib (SU5416) for further characterization of its effects on *CCM3* KO and WT cell proliferation and gene expression. Semaxinib is a known inhibitor of VEGFR2 [10], which is a major receptor of the VEGF signaling pathway and plays a critical role in regulating angiogenesis, vascular permeability, as well as migration, proliferation, and survival of ECs [19]. In CCM disease models, VEGF signaling has been described to be activated in ECs after CCM loss [5, 6, 50, 51]. However, He et al. also demonstrated that CCM3 deletion can destabilize VEGFR2 protein levels and impair VEGF‑induced signaling in ECs [13], highlighting the complex role of this pathway in CCM disease and the need for further research.

Our study shows that semaxinib selectively inhibited WT, but not *CCM3* KO cell proliferation in co-culture, and triggered different transcriptional responses in these cell types.

## Materials and methods

### Cell culture of induced pluripotent stem cells

The iPSC lines AICS-0054-091 (mTagRFP-T-labeled) and AICS-0036-06 (mEGFP-labeled) as part of the Allen Cell Collection (Coriell Institute, Camden, NJ, USA) were used in this study. *CCM3* KO clones were derived from the AICS-0036-06 iPSC line after CRISPR/Cas9 genome editing as described previously [33]. IPSCs were cultured in growth factor-reduced Matrigel-coated (#356231, Corning, NY, USA) 6-well plates using Essential 8 Flex medium (#A2858501, Thermo Fisher Scientific, Waltham, MA, USA) at 37 °C and 5% CO₂. Passaging was performed every three to four days using 0.5 mM EDTA. IPSC cultures were regularly tested for mycoplasma contamination by PCR and confirmed negative.

### Differentiation of iPSCs into endothelial cells

IPSCs were differentiated into ECs using the STEMdiff Endothelial Differentiation Kit (#08005, STEMCELL Technologies, Vancouver, Canada) following the manufacturer’s instructions. IPSCs at 70–80% confluency were dissociated into a single-cell suspension using Accutase (#A1110501, Thermo Fisher Scientific) and seeded at a density of 1 × 10^5^ cells/well on Matrigel-coated 6-well plates in Essential 8 Flex medium supplemented with 10 µM ROCK inhibitor Y-27632 (#72304, STEMCELL Technologies). On day 7 of the differentiation protocol, iECs were harvested with Accutase, resuspended in STEMdiff Endothelial Expansion Medium (#100-1218, STEMCELL Technologies), and seeded at a density of 1.3 × 10^5^ cells/well into 6-well plates coated with an animal component-free (ACF) cell attachment substrate for expansion. IECs were used for subsequent experiments at passages 1–3. The endothelial identity was confirmed by immunostaining for endothelial markers as previously described [29].

### Compound library screening

High-throughput screening of a cytokine inhibitor library was performed in co-cultures of AICS-0036 *CCM3* KO and AICS-0054 WT iEC co-cultures at a 1:4 ratio. DMSO-treated AICS-0036 WT/AICS-0054 WT and AICS-0036 *CCM3* KO/AICS-0054 WT iEC co-cultures were used as controls. Cells were seeded in 384-well plates coated with an ACF cell attachment substrate at a density of 1 × 10^3^ cells/well. Cells were maintained in EndoGRO-MV medium (#SCME004, Merck Millipore, Darmstadt, Germany) supplemented with 1 ng/ml fibroblast growth factor 2 (FGF-2) (#130-093-564, Miltenyi Biotec, Bergisch Gladbach, Germany). The TargetMol cytokine inhibitor library (#L3600, TargetMol Chemicals Inc., Boston, MA, USA; status 2022) was added two hours after seeding at a final concentration of 10 µM per well. The medium was replaced with fresh supplemented EndoGRO-MV medium containing the compounds on day 3. On day 6, plates were fixed with 4% paraformaldehyde (PFA) for 20 min at room temperature and stained with Hoechst 33342. Imaging was performed using the Operetta CLS High-Content Imaging System (PerkinElmer, Waltham, MA, USA) in non-confocal mode with a 5x objective.

### 2D proliferation and treatment studies

For 2D proliferation studies under different treatment conditions, AICS-0036 *CCM3* KO and AICS-0054 WT iEC monocultures or AICS-0036 *CCM3* KO/AICS-0054 WT co-cultures (ratio 1:4) were seeded in 96-well plates coated with ACF cell attachment substrate at a total cell density of 3–5 × 10³ cells/well and cultured in EndoGRO-MV medium supplemented with 1 ng/ml FGF-2 at 37 °C and 5% CO₂. After two hours of incubation, cells were treated with compounds as indicated. On day 3, medium containing the substances was refreshed. Cells were fixed with 4% PFA and stained with Hoechst 33342 at the indicated timepoints. Imaging and cell counting were performed with the Operetta CLS High-Content Imaging System in non-confocal mode with a 10x objective or the EVOS M5000 microscope (Thermo Fisher Scientific) with a 10x objective and ImageJ (FIJI v.1.54). The following compounds were used to treat the cell cultures: semaxinib (#HY-10374, MedChemExpress, NJ, USA), (Z)-semaxinib (#T2496, TargetMol), VEGFA (#100-20-50, Peprotech, Hamburg, Germany), bevacizumab (#HY-P9906, MedChemExpress), CH-223191 (#182705, Sigma-Aldrich), FICZ (#SML1489, Sigma-Aldrich), SC79 (#123871, Merck), PMA (#P1585, Sigma-Aldrich), MK-2206 (#HY-10358, MedChemExpress), and U0126 (#U120, Sigma-Aldrich). DMSO-treated cultures served as controls.

### Treatment of blood vessel organoids

To generate mosaic blood vessel organoids, mEGFP-tagged AICS-0036 *CCM3* KO iPSCs and mTagRFP-T-tagged AICS-0054 WT iPSCs were mixed at a 1:19 ratio. Organoids were differentiated using our established protocol as described previously [38]. From day 10 of differentiation, networks or organoids were treated with 10 µM semaxinib or DMSO (control) at each medium change (every two to three days). On day 17, organoids were fixed in 1% PFA for one hour at room temperature. Imaging and quantification of mEGFP fluorescence intensity were performed using the Operetta CLS High-Content Imaging System.

### VEGF ELISA

Cell culture supernatants of WT and *CCM3* KO iEC monocultures or *CCM3* KO/WT co-cultures were collected after three and six days of cultivation. Human VEGF levels were quantified using the LEGEND MAX Human VEGF ELISA Kit (#446507, BioLegend, San Diego, CA, USA) according to the manufacturer’s instructions. Briefly, standards and samples (measured in duplicates) were added to microplate wells pre-coated with a monoclonal antibody specific for human VEGF and incubated for two hours at room temperature. After washing, an enzyme-linked polyclonal antibody specific for VEGF was added, followed by substrate solution. After stopping of the reaction, the absorbance was measured at 450 nm and at 570 nm using a microplate reader.

### Immunofluorescence and flow cytometry analyses for VEGFR2

Staining was performed using the Immunofluorescence Application Solutions Kit (#12727, Cell Signaling Technology, Danvers, MA, USA) according to the manufacturer’s instructions. Cells were fixed in 4% PFA for 20 min at room temperature, washed with PBS, permeabilized with 0.1% Triton X-100 in PBS for 15 min prior to blocking, and then incubated with primary antibodies against VEGFR2 (#2479S, 1:200, 55B11, Cell Signaling Technology) or p-VEGFR2 (Tyr1175) (#2478S, 1:100, Cell Signaling Technology). After overnight incubation at 4 °C, samples were washed and incubated with anti-rabbit IgG Alexa Fluor 647 secondary antibody (#A21246, 1:200, Thermo Fisher Scientific). Nuclei were stained with Hoechst 33342. Analyses were performed using the Operetta CLS High-Content Imaging System in non-confocal mode with a 40x air objective for quantification or in non-confocal mode with a 63x water objective for representative immunofluorescence images.

For flow cytometry, 1 × 10⁶ cells of AICS-0036 *CCM3* KO and AICS-0054 WT iEC co-cultures (ratio 1:4) were seeded in T75 flasks and treated with 10 µM semaxinib or DMSO for six days as previously described. Cells were dissociated with Accutase and resuspended in PBS containing 1% BSA and 2 mM EDTA. VEGFR2 was stained with an Alexa Fluor 647 anti-human VEGFR2 antibody (#359909, BioLegend) at 5 µL per 1 × 10⁶ cells in 100 µL. Cells were incubated for 30 min at 4 °C in the dark, washed twice, and then immediately analyzed. All steps were performed on ice. Unstained cells were used as negative control. Flow cytometry was performed with a BD FACSAria III Cell Sorter (BD Biosciences, Franklin Lakes, NJ, USA) and data were analyzed with the FlowJo v10.10.1 software (BD Biosciences).

### FACS, RNA sequencing, and data analysis

To obtain sufficient RNA for expression analysis, a total of 1 × 10⁶ cells of AICS-0036 *CCM3* KO/AICS-0054 WT iEC co-cultures (ratio 1:4) were seeded in T75 culture flasks. Cells were cultured for four days and treated with 10 µM semaxinib or DMSO (control) as previously described. On day 4, cells were dissociated using Accutase and resuspended in PBS. Cell sorting was performed using a BD FACSAria III Cell Sorter (BD Biosciences). Sorted cells were immediately lysed in QuickExtract DNA Extraction Solution (#QE09050, Biosearch Technologies, Teddington, UK) or TRIzol Reagent (#15596018, Thermo Fisher Scientific) for DNA or RNA extraction. To confirm the purity of sorted cell populations, the extracted DNA was used for amplicon deep sequencing as described before [36]. RNA was purified using the Direct-zol RNA MiniPrep Plus Kit (#R2072, Zymo Research, Irvine, CA, USA) following the manufacturer’s instructions. RNA yield was quantified using the Qubit 4.0 Fluorometer and Qubit RNA HS Assay Kit (#Q32852, Thermo Fisher Scientific). RNA integrity was assessed on the 4150 TapeStation System (Agilent Technologies, Santa Clara, CA, USA) using the RNA ScreenTape Assay (#5067-5576, Agilent Technologies).

Poly(A)-enriched RNA-seq libraries were sequenced (2 × 150 bp) on an Illumina NovaSeq platform (Illumina, San Diego, CA, USA) by GENEWIZ (Leipzig, Germany). Raw sequencing reads were trimmed using Trimmomatic v.0.36 to remove adapter sequences and low-quality bases. Trimmed reads were then mapped to the Homo sapiens GRCh38 reference genome (ENSEMBL) using the STAR aligner v.2.5.2b. Unique gene hit counts were calculated using featureCounts from the Subread package v.1.5.2. Gene hit counts were used for downstream differential expression analysis. Comparison of gene expression between groups of samples was performed using DESeq2. The p-values and log2 fold changes were generated using the Wald test. Pre-ranked Gene Set Enrichment Analysis (GSEA) was performed on genes ranked by the DESeq2 Wald statistic using the human NABA MATRISOME gene set with the default settings. Gene Ontology (GO) Biological Process analyses were performed with ShinyGO V0.82.

### Statistical analysis

Quantification of cell counts was performed using the Operetta CLS High-Content Imaging System (PerkinElmer) or FIJI v.1.54 (ImageJ). R Studio and GraphPad Prism were used for statistical tests. Normality was assessed by the Shapiro-Wilk test. Data are presented as means and SD. Three to six biological replicates were carried out for each condition. In some cases, data were normalized to the DMSO control or semaxinib treatment conditions and expressed as a percentage. Normalization data are indicated in the figure legends. One- or two-way ANOVA with Dunnett’s or Tukey’s correction for multiple comparisons or two-tailed unpaired t-test were performed, and adjusted p-values or p-values < 0.05 were considered statistically significant, as described in the figure legends.

## Results

### Cytokine inhibitor library screening reveals compounds that modulate the tumor-like proliferation of *CCM3* KO cells

**Fig. 1:**
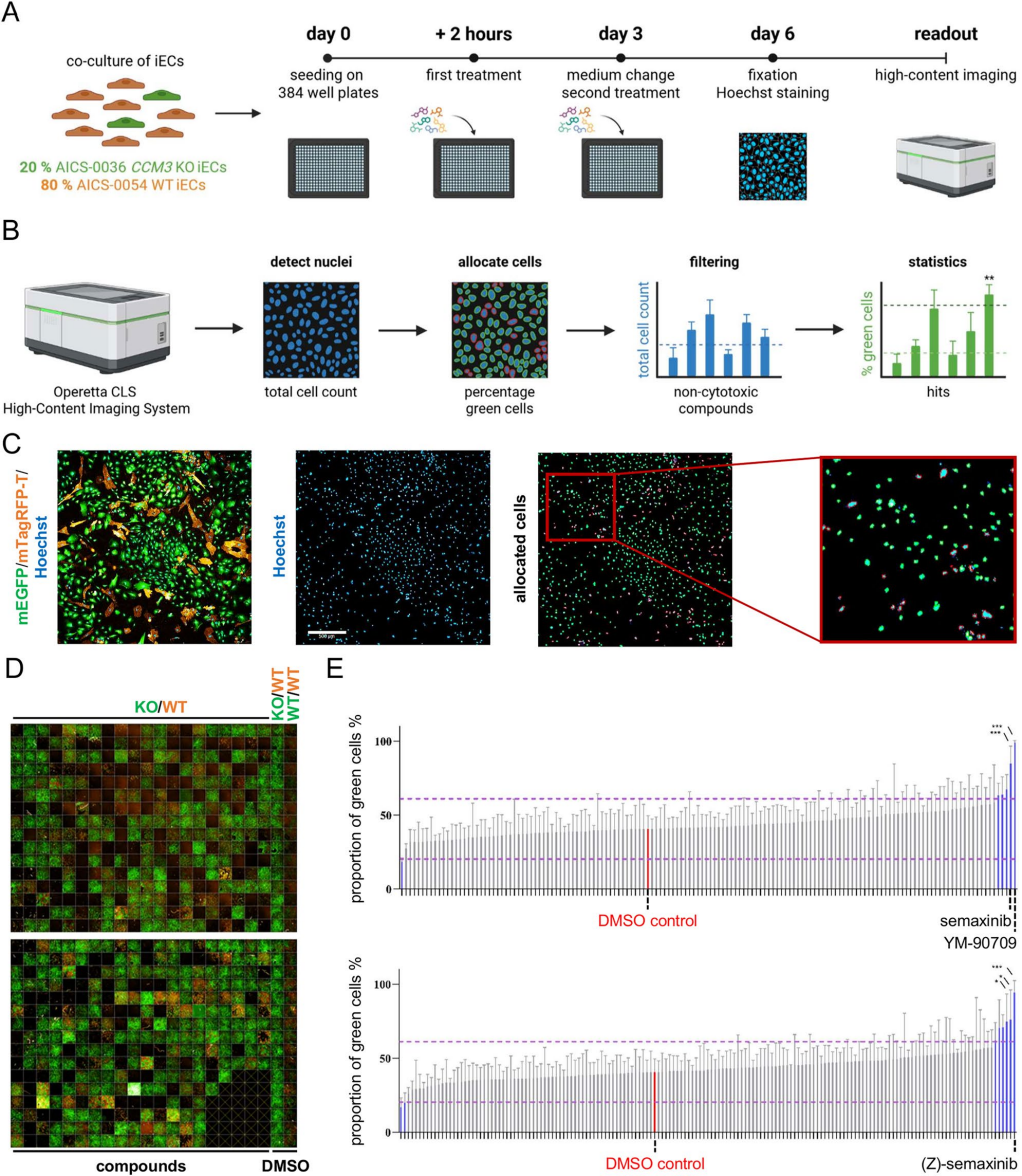
Screening of a cytokine inhibitor library in co-cultures of *CCM3* KO and WT iECs. **A** Scheme of the library screening approach (Created in BioRender. Pilz, R. (2026) https://BioRender.com/lhhhtxj). **B** Scheme of the image and data analysis strategy (Created in BioRender. Pilz, R. (2026) https://BioRender.com/o4wswi1). The Operetta CLS High-Content Imaging System was used to calculate the total number of nuclei, to allocate the cells to red or green fluorescence (colored outlines of the cells), and to quantify the percentage of green cells of the total cell count per well. The total cell count was used to exclude cytotoxic compounds. Then, statistical analysis was performed to identify hits. **C** Example of the readout strategy. The first two panels show fluorescence imaging of mEGFP (labeled cytoplasm of AICS-0036 cells), mTagRFP-T (labeled plasma membrane of AICS-0054 cells), and Hoechst 33342 (nuclei) (scale bar = 500 μm). The two panels on the right illustrate the software’s detection and allocation strategy, with an enlarged section for clarity. Nuclei of AICS-0036 cells are outlined in green, while nuclei of AICS-0054 cells are outlined in red. **D** Representative illustrations of the high-content imaging of plates from one experimental run. The library (compounds) was tested in co-cultures of *CCM3* KO (mEGFP) and WT (mTagRFP-T) iECs. DMSO-treated AICS-0036 *CCM3* KO/AICS-0054 WT and AICS-0036 WT/AICS-0054 WT co-cultures were used as controls (two columns on the right). **E** Results of the compound screening showing the percentage of green KO cells from the total cell count for each compound (bars) after excluding cytotoxic compounds. Data are presented as means and SD (*n* = 4). All conditions that showed a proportion of green KO cells higher or lower than 50% (purple lines) of the DMSO-treated KO/WT control (highlighted in red) are highlighted in blue. Statistical analysis was performed using one-way ANOVA with Dunnett’s correction (* = Padj < 0.05; *** = Padj < 0.001). All compound-treated conditions were compared against the DMSO-treated control

We employed an in vitro, two-dimensional HTS approach to analyze the effect of a cytokine inhibitor library on the tumor-like proliferation of *CCM3* KO cells in co-culture with WT cells. Co-cultures of iECs that are endogenously tagged with mEGFP (AICS-0036, *CCM3* KO) or mTagRFP-T (AICS-0054, WT) were treated with a compound library consisting of 613 small molecules targeting key cytokine and signaling pathways involved in vascular development, inflammation, and tumorigenesis. After fixation and Hoechst 33342 staining, the cultures were analyzed with high-content imaging using the Operetta CLS High-Content Imaging System (Fig. 1A). Taking advantage of the endogenous fluorescence tags of the cells and the nuclei staining, the system could accurately calculate the percentage of mEGFP-tagged KO cells in the culture (Fig. 1B, C). In a first step, a DMSO-treated WT/WT (mEGFP/mTagRFP-T) control was used to identify cytotoxic compounds. Substances that led to a total cell count below 80% of the WT/WT control were defined as cytotoxic [40] and excluded from statistical analysis. In the second step, all remaining treatments (309 out of 613 compounds) were compared to a DMSO-treated KO/WT control to identify compounds that significantly modulate the tumor-like proliferation of *CCM3* KO cells (Fig. 1B).

High-content imaging revealed noticeable differences in the proportion of mEGFP-tagged KO cells between the DMSO treated KO/WT and WT/WT control as well as between the treated conditions, which already became apparent at the visual level (Fig. 1D). The majority of compounds, however, did not lead to a significant change in the percentage of mEGFP-tagged KO cells compared to the DMSO treated KO/WT control with a mean value of 40.75% (Fig. 1E). Three compounds notably reduced the percentage of mEGFP-tagged KO cells: the BMP signaling agonist SB4 (16.86%), the EGFR/JAK3 inhibitor WHI-P154 (18.63%), and the ALK5 inhibitor SB 525334 (19.89%) (Additional File 1). However, these effects were not statistically significant. Five compounds significantly increased the percentage by more than 50% of the control. The most pronounced effects were observed for the IL-5 receptor antagonist YM-90709 (99.18%) and the VEGFR2 inhibitors semaxinib (85.01%) and (Z)-semaxinib (94.31%) (Fig. 1E, Additional File 1). Considering the contrasting observations in the literature regarding the role of VEGF/VEGFR2 signaling in CCM pathogenesis, we decided to further investigate the effect of semaxinib and (Z)-semaxinib on the proliferation of *CCM3* KO and WT ECs.

### Semaxinib has a dose-dependent and selective inhibitory effect on WT cells in co-culture

**Fig. 2:**
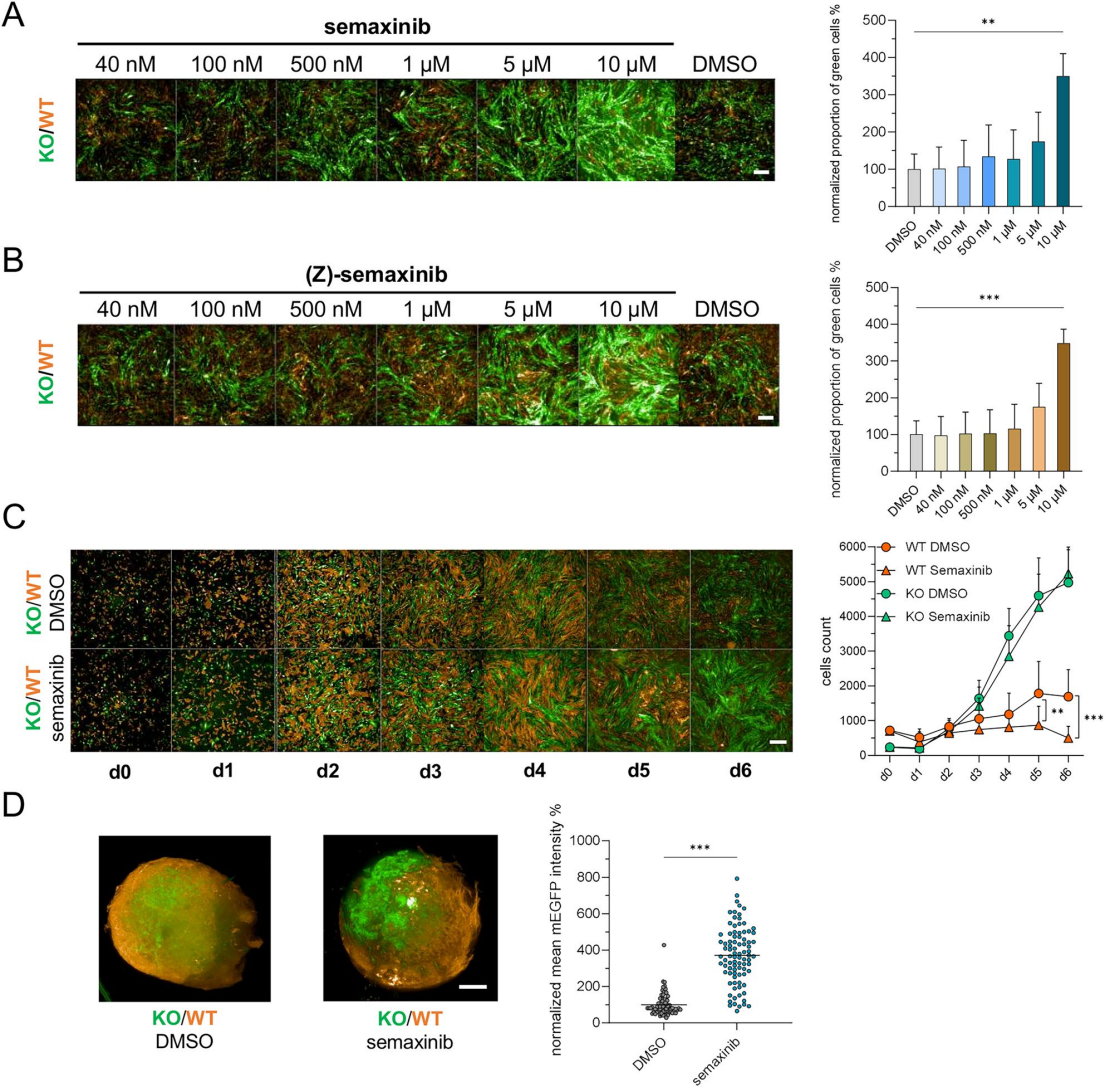
Semaxinib’s effect on *CCM3* KO and WT iEC proliferation in 2D co-culture and vascular organoids. **A**,** B** Co-cultures of *CCM3* KO (mEGFP) and WT (mTagRFP-T) iECs were treated with semaxinib (A) or (Z)-semaxinib (B) at various concentrations and analyzed after six days to assess the dose-dependent effects on proliferation. DMSO-treated co-cultures were used as controls. Representative fluorescence images (scale bar = 500 μm) and the proportion of *CCM3* KO cells normalized to the DMSO control and presented as means and SD (*n* = 3) are shown for both inhibitors. One-way ANOVA followed by Dunnett’s multiple comparisons test was used for statistical analysis (** = Padj < 0.01; *** = Padj < 0.001). **C** Co-cultures of *CCM3* KO and WT iECs were treated with 10 µM semaxinib or DMSO (control) and fixed on days 0, 1, 2, 3, 4, 5, and 6. Representative fluorescence images are shown (scale bar = 500 μm). The number of WT and KO cells was quantified using the Operetta CLS High-Content Imaging System and is displayed as means and SD (*n* = 6). Two-way ANOVA with Tukey’s post hoc test for multiple comparisons was used (** = Padj < 0.01; *** = Padj < 0.001). **D** Representative images of mosaic vascular organoids generated from co-cultures of *CCM3* KO and WT iPSCs at a ratio of 1:19 are shown after treatment with DMSO or semaxinib (scale bar = 200 μm). Mean mEGFP intensity normalized to the DMSO control is displayed as individual data points and means (*n* = 75–88). Statistical significance was assessed using a two-tailed unpaired t-test (*** = *P* < 0.001)

To further characterize the effect of semaxinib and the isomeric form (Z)-semaxinib on the proliferation of *CCM3* KO and WT iECs in co-cultures, we first conducted a dose-response study, which showed that the proportion of mEGFP-tagged KO cells increased in a dose-dependent manner similarly for both inhibitors (Fig. 2A, B). The strongest effect was observed for 10 µM of the inhibitors, while it was less pronounced for a concentration of 5 µM. Based on these results and other in vitro studies using semaxinib [16, 41, 43], we decided to apply 10 µM of semaxinib in the following experiments. We next analyzed the change in cell number for KO and WT cells in co-culture over a period of six days. While the cell number of mTagRFP-T-tagged WT cells increased over time for the DMSO-treated condition, it plateaued and even decreased after reaching full confluency under semaxinib treatment (Fig. 2C). In contrast, the proliferative capacity of mEGFP-tagged KO cells was not restricted. These results show that the increased proliferative advantage of KO cells under semaxinib treatment is due to inhibition and increased death of WT cells. Notably, semaxinib’s effect on proliferation could not only be observed for two-dimensional co-cultures of iECs, but also in three-dimensional mosaic vascular organoids that were differentiated from co-cultures of mEGFP-tagged *CCM3* KO and mTagRFP-T-tagged WT iPSCs according to our recently established protocol [38]. Treatment with semaxinib led to a strong increase in the mean mEGFP intensity compared to the DMSO-treated control (Fig. 2D).

### Semaxinib does not affect proliferation of monocultures despite differences in VEGFR2 expression

**Fig. 3:**
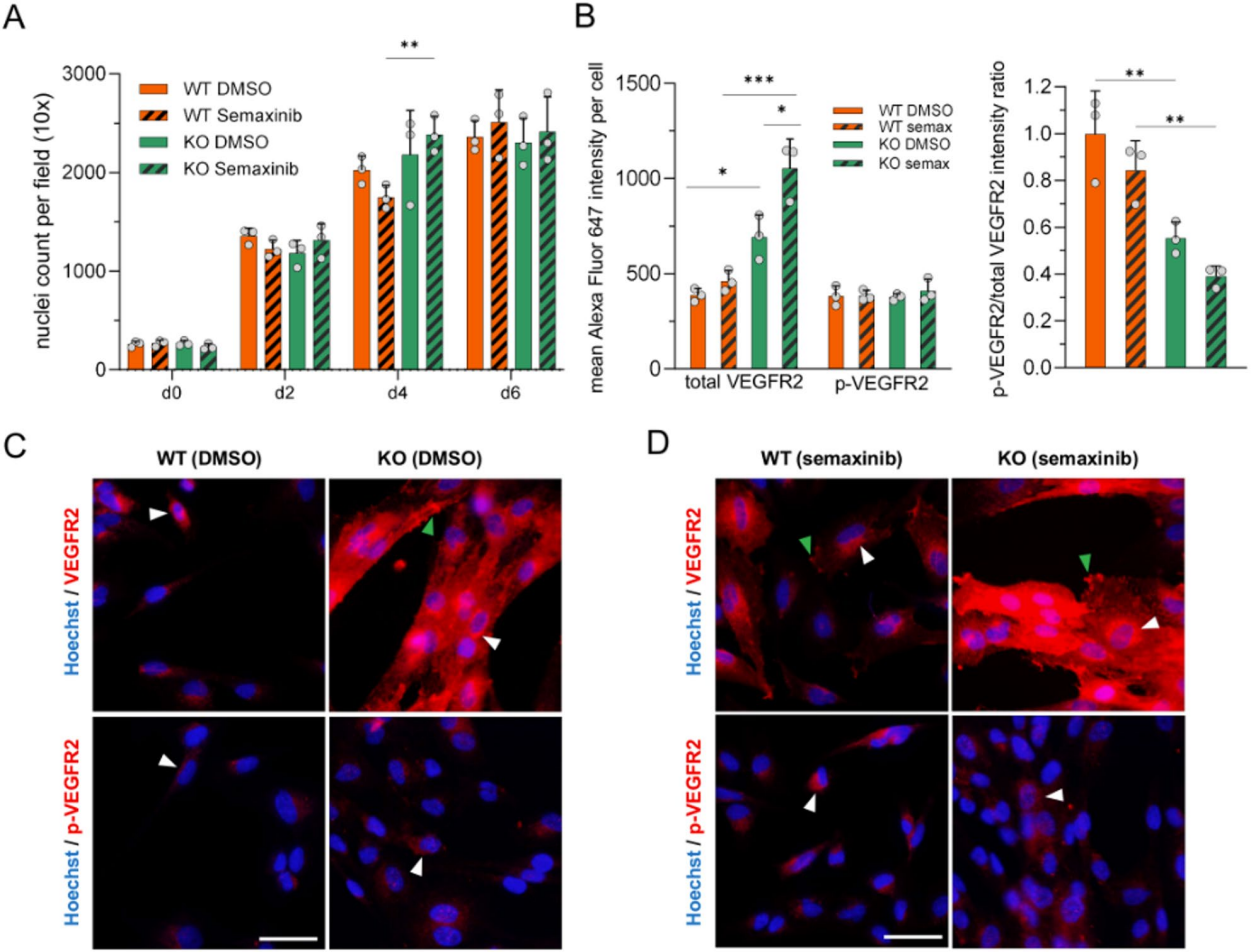
Proliferation and VEGFR2 expression analyses in *CCM3* KO and WT iEC monocultures. **A** Monocultures of AICS-0036 *CCM3* KO and AICS-0054 WT iECs were fixed on days 0, 2, 4, and 6. Nuclei counts were determined using FIJI v.1.54 (ImageJ) after staining with Hoechst 33342. Data are presented as means and SD (*n* = 3). Two-way ANOVA with Tukey’s post hoc test for multiple comparisons was used for statistical analysis (** = Padj < 0.01). **B–D** Total VEGFR2 and p-VEGFR2 (Tyr1175) expression was assessed by immunofluorescence staining in monocultures of *CCM3* KO and WT iECs after DMSO or 10 µM semaxinib treatment. **B** Quantification of the mean (p)-VEGFR2 staining intensity per cell and corresponding p-VEGFR2/total VEGFR2 ratios. Data are presented as means and SD (*n* = 3). Statistical significance was assessed using two-way ANOVA with Tukey’s post hoc test for multiple comparisons (* = Padj < 0.05; ** = Padj < 0.01; *** = Padj < 0.001). **C**, **D** Representative immunofluorescence images of total and p-VEGFR2 staining (scale bar = 50 μm). Arrows indicate examples of perinuclear (white) and membrane (green) staining

To determine whether the observed effect on proliferation was specific to the co-culture condition, monocultures of *CCM3* KO and WT iECs were treated with semaxinib. Cell numbers were quantified over time at day 0, 2, 4, and 6 post-treatment. Overall, no major differences were found between either the treatment conditions or the genotypes (Fig. 3A). This confirmed that *CCM3* inactivation does not increase proliferation by itself. *CCM3* KO iECs only show highly increased proliferation under co-culture conditions. Particularly, these findings also highlight that semaxinib is not directly cytotoxic. WT iECs become sensitive to this treatment only in co-culture.

We next asked whether expression of total and phosphorylated VEGFR2 (p-VEGFR2) correlates with the observed proliferative behavior. In DMSO control conditions, immunofluorescence staining revealed markedly higher total VEGFR2 expression in KO compared to WT cells, while p-VEGFR2 staining intensity was not different between genotypes (Fig. 3B, C). Interestingly, the EndoGRO-MV growth medium did not contain any VEGFA and WT and KO iECs did not secrete detectable amounts of human VEGF into the cell culture medium, as measured by an ELISA (Additional file 2). *CCM3* KO cells thus upregulate VEGFR2 even though no VEGF is present as a ligand in the medium. Accordingly, no abnormal constitutive activation of the receptor was observed in KO cells. In semaxinib conditions, total VEGFR2 staining intensity was higher compared to DMSO treatment, especially in KO cells. Again, no differences in p-VEGFR2 staining intensity could be observed (Fig. 3B, D). Consequently, the p-VEGFR2/total VEGFR2 intensity ratio was slightly decreased under semaxinib treatment for both genotypes, and was lower in KO compared to WT cells in both treatment conditions (Fig. 3B).

Taken together, these results suggest the following: (1) CCM3 loss increases VEGFR2 expression, (2) WT and KO iECs proliferate independently from VEGFR2-associated signaling in monoculture, and (3) Semaxinib treatment leads to further accumulation of inactive VEGFR2.

### Semaxinib’s effect in co-culture does not interfere with signaling induced by extracellular VEGFA

**Fig. 4:**
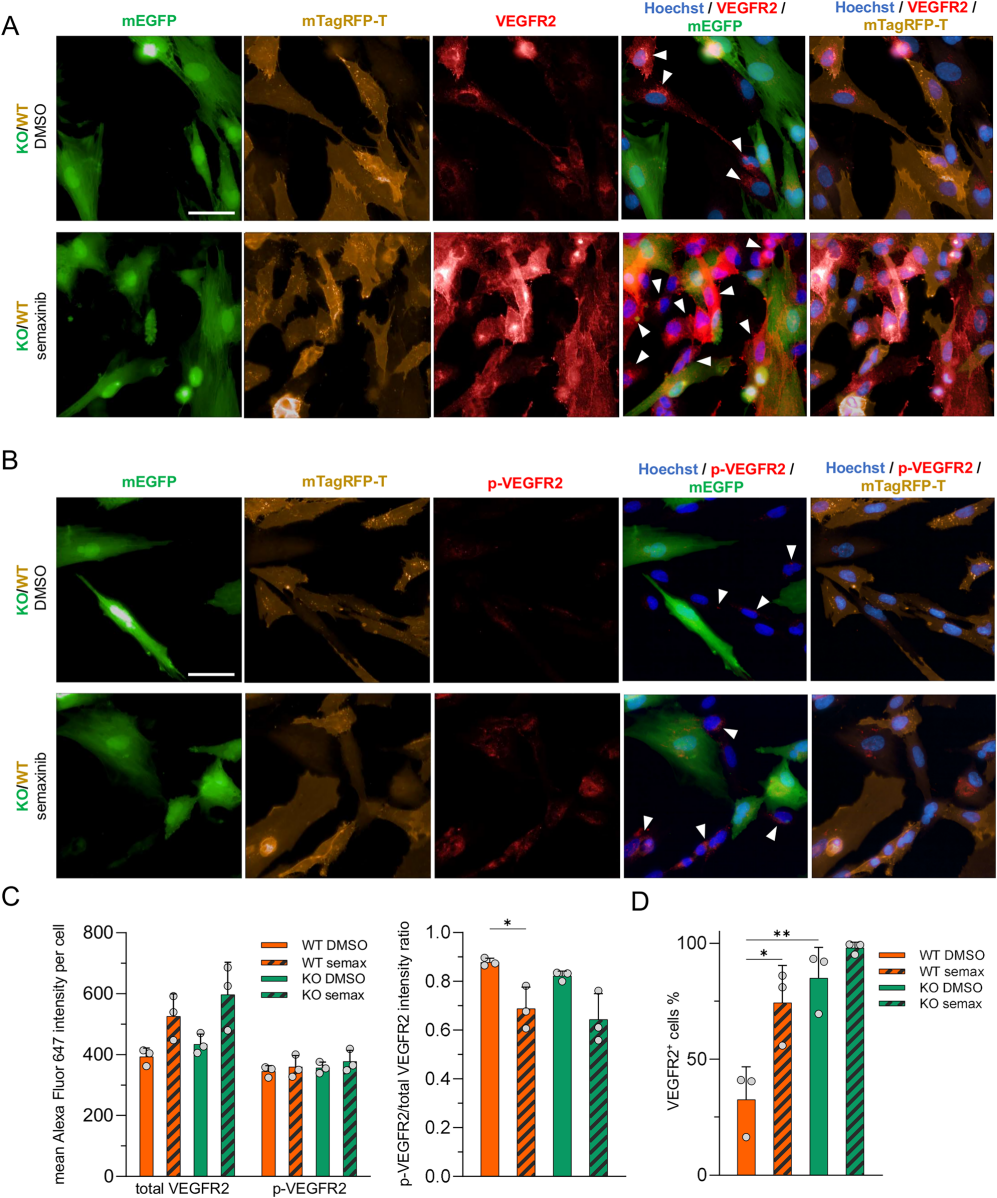
VEGFR2 expression analyses in *CCM3* KO/WT iEC co-cultures. **A–C** Total VEGFR2 and p-VEGFR2 (Tyr1175) expression was assessed by immunofluorescence staining in co-cultures of *CCM3* KO (mEGFP) and WT (mTagRFP-T) iECs after DMSO or 10 µM semaxinib treatment. **A**, **B** Representative immunofluorescence images of total and p-VEGFR2 staining (scale bar = 50 μm). White arrows indicate examples of WT cells expressing (p)-VEGFR2. **C** Quantification of the mean (p)-VEGFR2 staining intensity per cell and corresponding p-VEGFR2/total VEGFR2 ratios. Data are presented as means and SD (*n* = 3). Statistical significance was assessed using two-way ANOVA with Tukey’s post hoc test for multiple comparisons (* = Padj < 0.05). **D** Flow cytometry analysis of co-cultures stained for VEGFR2. The proportion of VEGFR2-positive (VEGFR2^+^) cells is shown for each WT and KO cells after DMSO or semaxinib treatment. Data are presented as means and SD (*n* = 3). Statistical significance was assessed using two-way ANOVA with Tukey’s post hoc test for multiple comparisons (* = Padj < 0.05, ** = Padj < 0.01)

Following up, we investigated whether (p)-VEGFR2 expression of KO and WT iECs changes under co-culture conditions. Although total and p-VEGFR2 staining intensities showed the same trend as in monoculture, differences between genotype and treatment conditions were not significant (Fig. 4A-C). We additionally performed flow cytometry analysis of co-cultures to more reliably differentiate between KO and WT cells. This confirmed that a significant higher proportion of KO compared to WT cells were positive for VEGFR2 and that semaxinib treatment increased the proportion of VEGFR2^+^ cells for both genotypes (Fig. 4D). These data suggest that co-culture conditions do not significantly alter the (p)-VEGFR2 expression of WT and KO cells.

**Fig. 5:**
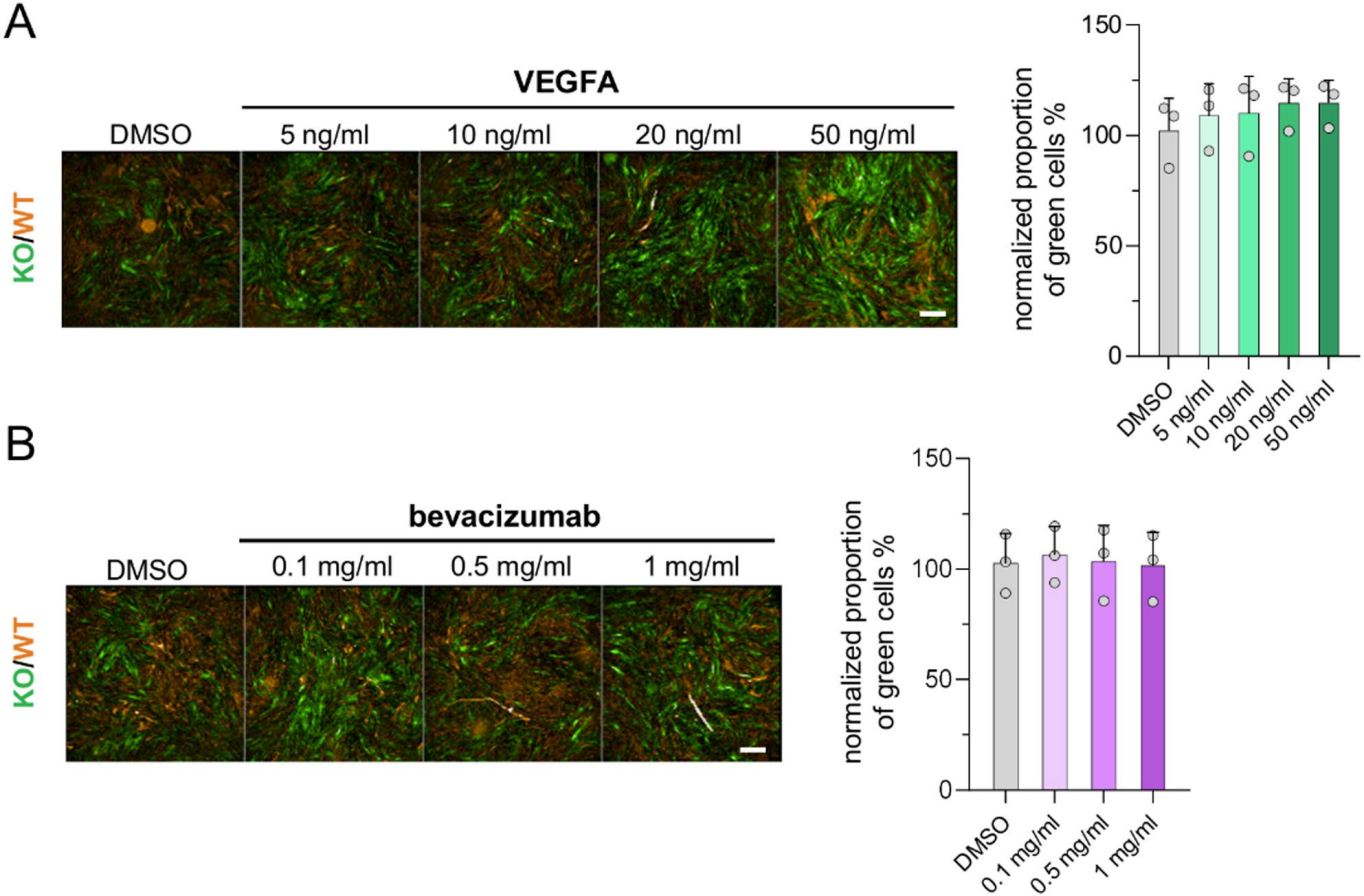
Proliferation analyses in *CCM3* KO/WT iEC co-cultures under VEGFA or bevacizumab treatment. **A**,** B** Co-cultures of *CCM3* KO and WT iECs were treated with different concentrations of vascular endothelial growth factor A (VEGFA) (**A**) or bevacizumab (**B**) and analyzed after six days to evaluate dose-dependent effects on cell proliferation. DMSO-treated co-cultures were used as controls. Representative fluorescence images (scale bar = 500 μm) and proportion of *CCM3* KO cells normalized to the DMSO control and presented as means and SD (*n* = 3) are shown. One-way ANOVA followed by Dunnett’s multiple comparisons test was used for statistical analysis

To further investigate whether interference with VEGF-induced signaling is involved in semaxinib’s effect on cell proliferation in co-culture, we tested the impact of VEGFA supplementation and VEGFA neutralization using the monoclonal antibody bevacizumab. The modulation of extracellular VEGFA levels by VEGFA supplementation at different concentrations did not notably affect co-culture dynamics (Fig. 5A). Similarly, treatment with bevacizumab also did not lead to changes in the proportion of KO cells (Fig. 5B). In line with this, no secretion of VEGF was observed in co-cultures either (Additional file 2). Therefore, the sensitivity of WT iECs to semaxinib treatment in co-culture could be due to an increased dependence on intracrine VEGF signaling for survival, or to other effects of the treatment that are not directly connected to VEGF signaling. In contrast, KO cells maintain their proliferative capacity.

### RNA-seq reveals differential responses of *CCM3* KO and WT cells to semaxinib treatment

**Fig. 6:**
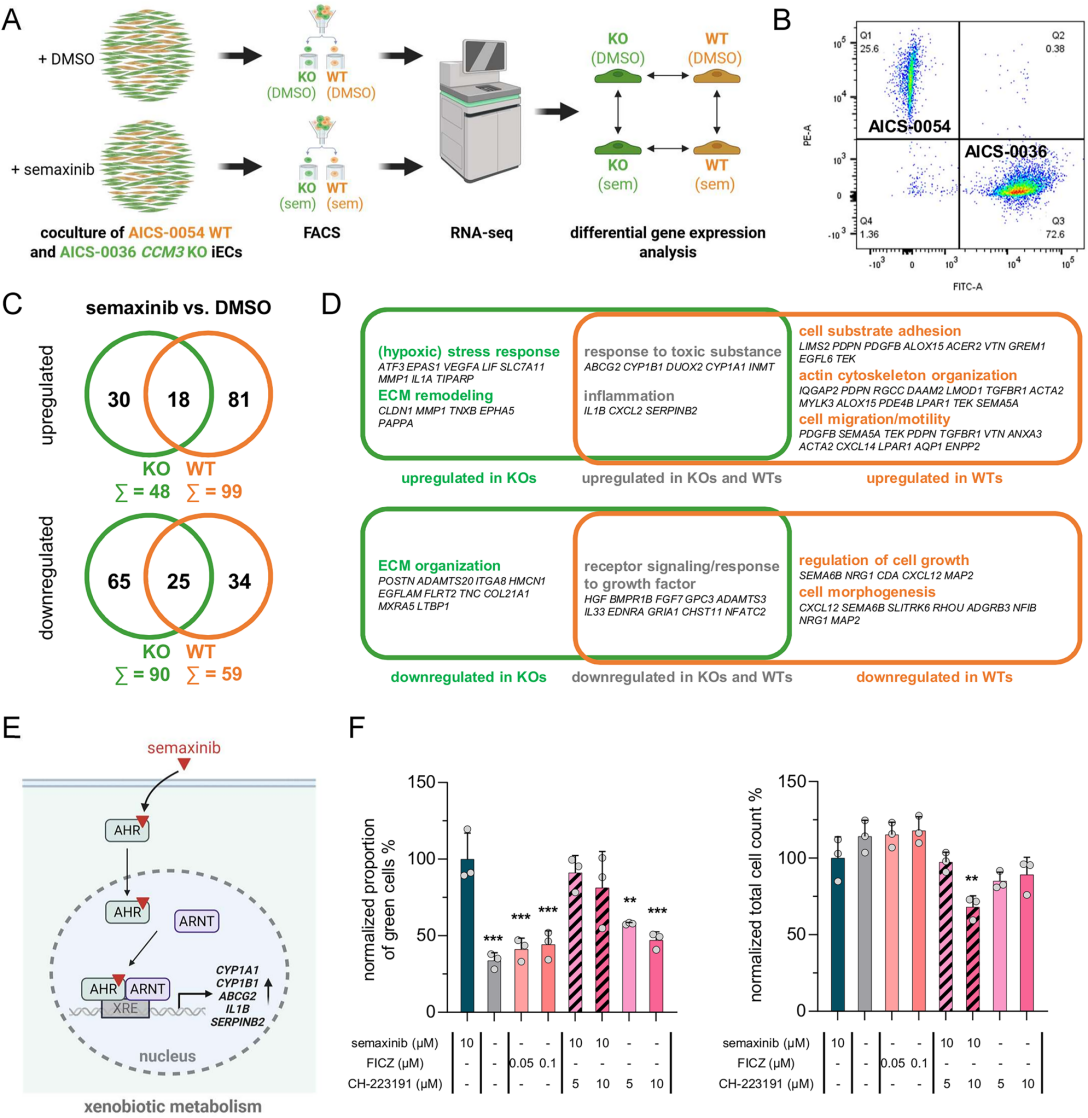
Semaxinib’s effect on the gene expression signature of *CCM3* KO and WT iECs in co-culture. **A** Scheme of the experimental procedure (Created in BioRender. Pilz, R. (2026) https://BioRender.com/kfh9xxe). sem = semaxinib. **B** Fluorescence-activated cell sorting (FACS) resulted in distinct populations of AICS-0036 *CCM3* KO (mEGFP-tagged, FITC) and AICS-0054 WT (mTagRFP-T-tagged, PE) cells after co-culture. **C**,** D** Number of genes that are deregulated only in KO or WT cells or in both genotypes after semaxinib treatment shown as a Venn diagram (**C**) and associated biological processes as determined by gene ontology analysis (**D**). **E** Scheme of AhR signaling activation by semaxinib (Created in BioRender. Pilz, R. (2026) https://BioRender.com/vxfv30o). **F** Co-cultures of *CCM3* KO and WT iECs were treated with semaxinib, the AhR agonist FICZ, and the AhR antagonist CH-223191 as indicated. Proportion of *CCM3* KO cells and total cell count each normalized to the semaxinib-only treated condition and presented as means and SD (*n* = 3) are shown. One-way ANOVA followed by Dunnett’s multiple comparisons test was used for statistical analysis, comparing each treatment to the semaxinib-only treated condition (first column) (** = Padj < 0.01; *** = Padj < 0.001)

To study the semaxinib-induced changes of the transcriptional signatures of WT and *CCM3* KO iECs in co-culture, we performed RNA sequencing (RNA-seq). Taking advantage of their different fluorescence labels, WT and *CCM3* KO iECs were separated by fluorescence-activated cell sorting (FACS) after co-culture (Fig. 6A, B). FACS and next-generation sequencing confirmed the high purity of the sorted cell populations (Fig. 6B, Additional File 3). Semaxinib treatment led to upregulation of 48 genes and downregulation of 90 genes in *CCM3* KO iECs. In contrast, WT iECs upregulated 99 genes and downregulated 59 genes (Fig. 6C, Additional File 4). The common response of both genotypes included increased expression of genes associated with drug efflux or metabolization (*ABCG2*, *CYP1A1*, *CYP1B1*, *DUOX2*) and inflammation (*IL1B*, *CXCL2*, *SERPINB2*) (Fig. 6D). This indicates that semaxinib not only acts as a VEGFR2 inhibitor, but also activates signaling via the aryl hydrocarbon receptor (AhR) (Fig. 6E) [22, 25]. As this was observed in both WT and KO cells, altered AhR signaling is unlikely to be the cause of semaxinib’s effect on the proliferation in co-culture. Consistently, treatment with the AhR agonist FICZ did not show the same effect as semaxinib, and simultaneous treatment with semaxinib and the AhR antagonist CH-223191 had no significant effect compared to the condition treated with semaxinib alone (Fig. 6F). Commonly downregulated genes in KO and WT after semaxinib treatment play a role in receptor signaling and response to growth factors (e.g., *HGF*, *BMPR1B*, *FGF7*, *EDNRA*, *GRIA1*) (Fig. 6D). The relatively small overlap of differentially expressed genes (DEGs) in KO and WT iECs highlights that these cells respond very differently to semaxinib treatment in co-culture. WT, but not *CCM3* KO iECs, deregulate processes that are related to cell adhesion, cytoskeleton organization, cell migration, motility, growth regulation, and morphogenesis, which is consistent with a blockage of the VEGFR2 signaling axis (Fig. 6D). These data support our observation that WT iECs become sensitive to VEGFR2 inhibition in co-culture.

In contrast, KO iECs showed no direct signs of VEGFR2 inhibition, but appear to activate a hypoxic-like stress response (Fig. 6D). This is supported by the upregulation of *EPAS1* (coding for the hypoxia-inducible factor 2 alpha HIF-2α), as well as the *VEGFA*, *ATF3*, *LIF*, *SLC7A11*, *MMP1*, *IL1A*, and *TIPARP* genes, which can be induced under hypoxic conditions. Interestingly, *TIPARP* has also been described as an AhR signaling target gene, which is involved in a negative feedback loop regulating AhR activity [21]. Moreover, extracellular matrix (ECM) remodeling processes seem to be activated as a response of KO iECs to semaxinib, including upregulated (*MMP1*, *TNXB*, *EPHA5*, *PAPPA*, *CLDN1*) and multiple downregulated ECM-associated genes (e.g., *POSTN*, *ADAMTS20*, *ITGA8*, *HMCN1*, *COL21A1*, *MXRA5*, *LTBP1*), indicating altered matrix deposition, signaling, and adhesion (Fig. 6D).

### *CCM3* KO cells deregulate extracellular matrix organization and show a mitotic gene expression signature both under control and semaxinib conditions

**Fig. 7:**
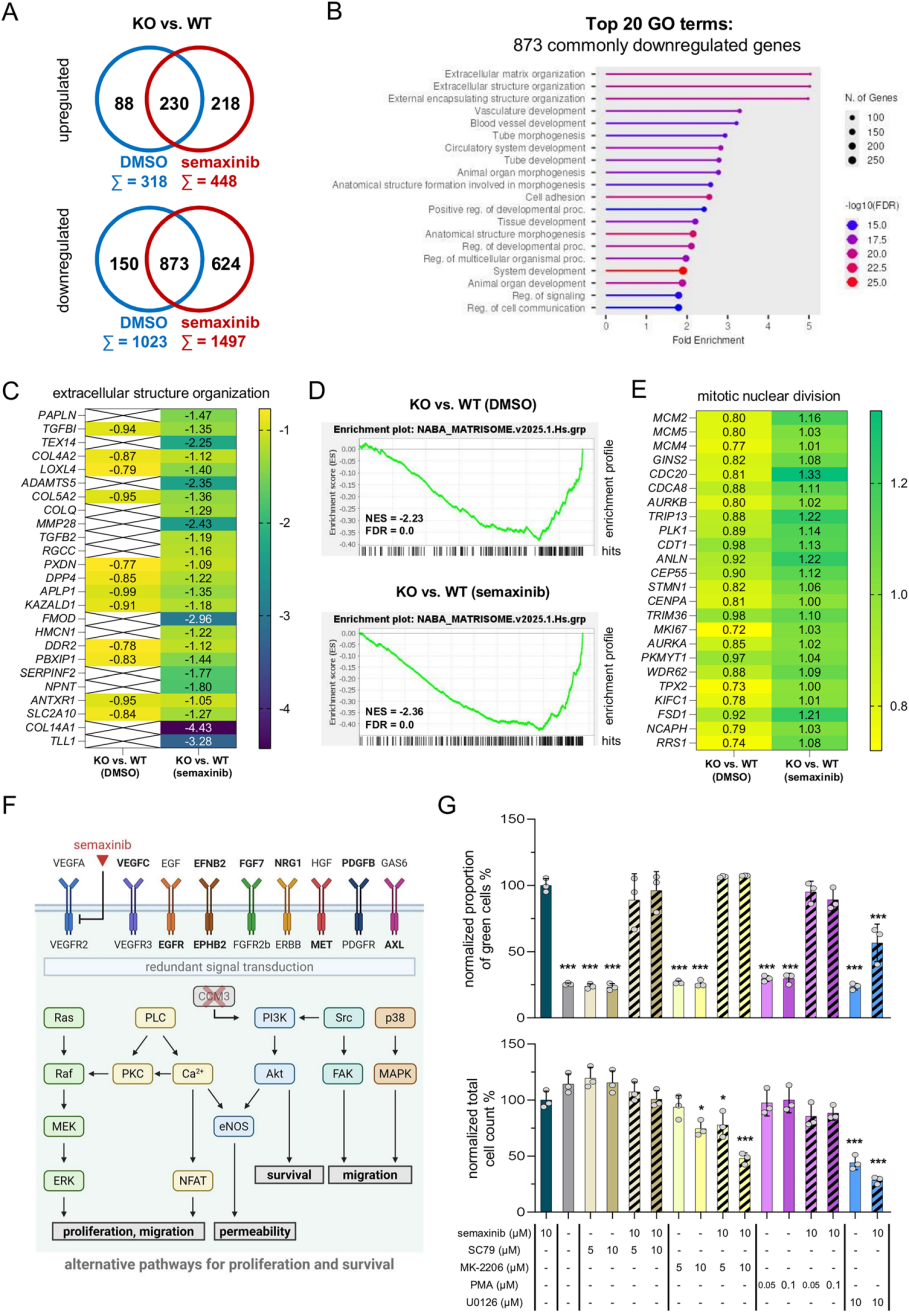
Expression differences between *CCM3* KO and WT iECs in co-culture under DMSO or semaxinib treatment. **A** Number of genes that are deregulated in KO cells compared to WT cells only under DMSO or semaxinib treatment or in both treatment conditions shown as a Venn diagram. **B** Gene ontology (GO) biological processes analysis of genes downregulated in KO cells in both DMSO and semaxinib conditions shown as a lollipop chart. **C** Heatmap showing log2 fold changes of genes associated with extracellular matrix organization that are only significantly downregulated (log2FC ≤ -1, Padj < 0.05) in KO cells under semaxinib-treated conditions. **D** Gene Set Enrichment Analysis of the gene set NABA_MATRISOME for significantly deregulated genes in KO cells compared to WT cells under DMSO- and semaxinib-treated conditions. NES = Normalized Enrichment Score, FDR = False Discovery Rate. **E** Heatmap showing log2 fold changes of genes that are significantly deregulated in KO cells (Padj < 0.05) and associated with mitotic nuclear division for DMSO and semaxinib-treated conditions. **F** Scheme of the main VEGFR2 signaling axes and redundant signal transduction through receptor tyrosine kinases (Created in BioRender. Pilz, R. (2026) https://BioRender.com/ud66y09). Corresponding genes of components marked in bold are upregulated in KO cells compared to WT cells under semaxinib- and partly already under DMSO-treated conditions. **G** Co-cultures of *CCM3* KO and WT iECs were treated with semaxinib, the Akt activator SC79, the Akt inhibitor MK-2206, the PKC activator PMA, and the MEK1/2 inhibitor U0126 as indicated. Proportion of *CCM3* KO cells and total cell count each normalized to the semaxinib-only treated condition and presented as means and SD (*n* = 3) are shown. One-way ANOVA followed by Dunnett’s post hoc test was performed, comparing each treatment to the semaxinib-only treated condition (first column) (* = Padj < 0.05; *** = Padj < 0.001)

After focusing on the semaxinib-induced transcriptional changes for each genotype, we next compared gene expression between the genotypes under control and treatment conditions to uncover the effects of the *CCM3* KO in co-culture and the mechanisms that could explain the different responses of WT and KO iECs to semaxinib. This revealed that ECM-associated processes are particularly deregulated. Genes that showed a significantly lower expression in KO cells under both DMSO and semaxinib conditions (*n* = 873, Fig. 7A) are enriched for ECM structure and organization (Fig. 7B, Additional File 4). These include, in particular, collagens or collagen components (*COL11A1*, *COL5A3*, *COL4A4*, *COL16A1*, *COL9A3*, *COL1A1*, *COL5A1*, *COL8A1*, *COL1A2*, *COL3A1*, *COL8A2*, *COL4A1*, *COL11A2*, *COL15A1*, *COL12A1*) and metalloproteinases of the ADAMTS family (*ADAMTS2*, *ADAMTS10*, *ADAMTS16*, *ADAMTS1*, *ADAMTS13*, *ADAMTS15*). Notably, under semaxinib condition, further ECM-associated genes are significantly downregulated in KOs compared to WTs (Fig. 7C). These comprise collagens (*COL14A1*, *COL4A2*, *COL5A2*), enzymes involved in ECM crosslinking and remodeling (*LOXL4*, *PXDN*, *MMP28*, *ADAMTS5*), as well as matricellular proteins (*FMOD*, *NPNT*). The alteration of the matrisome in WT and *CCM3* KO iEC co-cultures, which is even more pronounced after semaxinib treatment, is also illustrated by Gene Set Enrichment Analysis (Fig. 7D). Besides deregulation of ECM-associated processes, a distinct mitotic gene expression signature, even after semaxinib treatment, was noticeable for *CCM3* KO iECs. This comprises core mitotic regulators and proliferation markers (e.g., *MCM4/5*, *GINS2*, *CDC20*, *PLK1*,* AURKB*, *CENPA*, *TPX2*) that are overexpressed in KO cells in both DMSO and semaxinib conditions (Fig. 7E). In addition, further genes that are known to have an oncogenic or pro-survival function, or that are overexpressed in various cancers, are also upregulated in KO iECs in semaxinib and partly already in DMSO conditions (e.g., *BCL2*, *NSD2*, *FOXM1*, *HJURP*, *UHRF1*, *HMGA1*, *MECOM*, *MYBL1*, *MIR17HG*, *SLC7A11*, *NT5E*, *TSPAN8*, *FAM83D*). Similarly, several genes that have been described to have tumor suppressive functions display a lower expression in KO compared to WT iECs under semaxinib treatment (e.g., *SOX6*, *NDRG2*, *CRABP2*, *RSPO2*, *DAPK1*, *GADD45G*, *FOXO1*). Finally, several growth factors and receptor tyrosine kinases are overexpressed in KO cells in semaxinib and partly already in DMSO conditions (e.g., *VEGFC*, *EGFR*, *EFNB2*, *EPHB2*, *FGF7*, *NRG1*, *MET*, *PDGFB*, *AXL*). This may point to redundant signal transduction, for example, via the MEK/ERK or PI3K/Akt pathway, that KO iECs could exploit to maintain their proliferative capacity (Fig. 7F). Treatment of co-cultures with activators or inhibitors of these pathways, however, did not change co-culture dynamics (Fig. 7G). Only simultaneous treatment with semaxinib and 10 µM of the MEK1/2 inhibitor U0126 led to a significant reduction in the proportion of KO cells compared to the condition treated with semaxinib alone. However, a markedly reduced total cell count was observed, suggesting a cytotoxic effect (Fig. 7G). Treatment with lower concentrations of U0126, on the other hand, did not alter the co-culture dynamics (Additional file 5).

Together, these data indicate increased ECM remodeling as well as pro-survival and proliferative signaling in *CCM3* KO compared to WT cells. Together with a hypoxic stress response and activated drug metabolization, these processes may play an important role for KO cells to maintain their proliferative capacity in co-culture with WT cells under semaxinib treatment.

## Discussion

The abnormal expansion of CCM3-deficient ECs in co-culture with WT ECs is one of the most striking observations that emphasize the role of cancer-like mechanisms in CCM formation [33, 38]. A better understanding of this aberrant behavior and of the complex interactions between KO and WT cells is essential for the development of new CCM therapies.

Using a cytokine inhibitor library, we found that semaxinib, a known small molecule inhibitor of VEGFR2 that blocks its phosphorylation [10] and has reached phase III clinical trials for cancer treatment [12], suppressed WT cell proliferation in co-culture, while *CCM3* KO cells maintained their proliferative capacity. The contribution of VEGF/VEGFR2 signaling in CCM disease remains controversial, as both activation [5, 6, 50, 51] and impairment [13] of the pathway have been reported following CCM loss. As previous studies have shown for CCM3-deficient ECs [35, 36, 50], we noted markedly higher VEGFR2 expression in *CCM3* KO compared to WT iECs. However, we did not find evidence for increased receptor activation. Even higher VEGFR2 expression was observed under semaxinib conditions in our study. This could be explained by a previous observation that inhibition of VEGFR2 kinase activity by semaxinib blocks the ubiquitination and lysosomal degradation of activated VEGFR2, leading to accumulation of the receptor [8]. Notably, VEGFR2 inhibition in CCM mouse or human cell culture models led to lesion reduction or barrier stabilization (Additional File 6), yet its effects on cell proliferation were not directly examined. While differences in experimental strategies and model systems might account for some of the variation observed across studies, VEGFR2 signaling likely also has context-dependent functions. For example, semaxinib treatment decreased the number of lesions in a *Ccm1*^ieKO^ mouse model but did not limit lesion size, indicating that VEGF signaling is more relevant to lesion initiation than progression [5]. While our results do not exclude that VEGF signaling contributes to CCM lesion formation, they suggest that VEGFR2 inhibition is not effective to counteract the proliferative anomalies in established lesions. Instead, our results show that co-culture of WT and *CCM3* KO cells creates a niche in which only WT cells become sensitive to semaxinib treatment. However, co-culture dynamics were not changed by modulation of extracellular VEGFA levels. This could hint at an increased dependency of WT cells on intracrine VEGF signaling which has been described as essential for EC survival and vascular homeostasis [7, 18]. While the causes of the increased sensitivity of WT cells in co-culture remain to be elucidated, our RNA-seq data indicate that disturbance of the ECM microenvironment by KO cells alters the mechanical and adhesive properties of the co-culture, which may change mechanotransduction pathways and increase the dependency of WT cells on VEGFR2 signaling for survival and adhesion [17, 19, 23]. Notably, loss of ECM signaling can lead to anoikis, a form of programmed cell death, and resistance to anoikis can be mediated by compensatory activation of oncogenes and receptor tyrosine kinase (RTK) signaling [15, 28].

Our RNA-seq analysis further suggests that semaxinib exerts effects beyond VEGFR2 inhibition by activating AhR signaling. Consistent with previous reports identifying semaxinib as a potent AhR agonist [22, 25], we observed strong induction of AhR target genes, including those coding for the xenobiotic metabolizing enzymes CYP1A1 and CYP1B1, in both WT and *CCM3* KO iECs. These findings highlight that AhR pathway activation should also be considered when interpreting semaxinib responses. Interestingly, enhanced drug metabolization and efflux upon AhR activation may partly explain why semaxinib did not inhibit KO cell proliferation in co-culture. For example, the ABCG2 efflux transporter, an AhR target linked to multidrug resistance [42, 44], was overexpressed in KO iECs compared to WT iECs under semaxinib treatment. Likewise, induction of *TIPARP*, a negative regulator of AhR signaling [14], may further protect KO iECs from AhR-mediated stress [14, 21]. However, since AhR activation occurred in both genotypes, it cannot fully explain the differential response to semaxinib. Accordingly, treatment with an AhR agonist or antagonist did not change co-culture dynamics. Instead, our transcriptomic data indicate that additional mechanisms in KO cells sustain proliferation under semaxinib treatment in co-culture. These may involve a combination of ECM remodeling, alternative angiogenic and hypoxia-driven signaling which are known strategies exploited by tumor cells to evade anti-angiogenic therapies [19, 30, 37]. *CCM3* KO cells activated a transcriptional stress response resembling hypoxia, including upregulation of *EPAS1*, *ATF3*, *VEGFA*, *LIF*, and *SLC7A11* which have been associated with tumor progression [31, 47, 48]. Moreover, semaxinib-treated KO cells may bypass VEGFR2 inhibition by exploiting alternative growth factor and receptor tyrosine kinase pathways which share common downstream signaling via, for example, MEK/ERK or PI3K/Akt. This is suggested by the upregulation of *VEGFC*, *EGFR*, *NRG1*, *EFNB2*, *EPHB2*, *FGF7*, *MET*, *PDGFB*, and *AXL*. Activation or inhibition of PI3K/Akt or MEK/ERK signaling, on the other hand, did not rescue WT or inhibit KO cell proliferation in co-culture. However, as the treatments in co-culture do not specifically target one cell type, but affect both WTs and KOs, these observations should be interpreted with caution. Moreover, the survival advantage of KOs is likely due to a combination of different processes and not just one specific pathway.

Finally, our RNA-seq data not only revealed differential responses to semaxinib treatment but also highlighted ECM remodeling processes to play a central role in shaping co-culture dynamics of KO and WT iECs. In cancer biology, ECM alterations promote tumor progression, for example, by an increased matrix stiffness, altered mechanosignaling, or the release of bioactive matrikines and matrix-bound growth factors that stimulate tumor proliferation, migration, and angiogenesis [46]. Consistent with this, *CCM3* KO iECs showed lower expression of ECM components and upregulation of metalloproteinases which supports the idea that an altered ECM contributes to CCM lesion progression and maturation [9]. In future studies, it would be particularly interesting to investigate whether and how these processes mediate the proliferation advantage of *CCM3* KO cells in co-culture. Methodologically, our EC co-culture assay, which reflects the mosaic nature of CCMs, is easy to handle, exploits a simple readout strategy, and allows targeting the KO/WT interaction in a scalable format. Together with the recently established high-throughput differentiation of more complex mosaic vascular organoids [38], human iPSC-based screening platforms might significantly enhance our mechanistic understanding of CCM pathogenesis and accelerate the drug identification process.

## Conclusions

Our study demonstrates that co-culture conditions render WT iECs sensitive to semaxinib. In contrast, *CCM3* KO cells may exploit hypoxia-like stress responses, activation of alternative growth signaling pathways, and ECM remodeling to maintain their survival advantage. These findings extend our understanding of non-cell-autonomous mechanisms and complex interactions between WT and KO ECs in CCM pathogenesis. Thus, our human iPSC-based co-culture assay represents a powerful and scalable platform to study cellular interactions and to identify novel therapeutic strategies.

## Supplementary Information

Below is the link to the electronic supplementary material.

Additional file 1. Results of the library screening. Proportion of green cells for each compound of the library and each run.

Additional file 2. Human VEGF ELISA for WT and CCM3 KO mono- and co-cultures. ELISA plate layout and absorbance measurements for WT and CCM3 KO mono- and co-cultures after cultivation for three or six days.

Additional file 3. Confirmation of the high purity of sorted cell populations after FACS of WT and CCM3 KO iEC co-cultures for RNA-seq analysis. Exemplary depiction of post-sorting (A) and next-generation sequencing (NGS) data (B) of the sorted cell populations. The NGS data is shown as excerpts from the SequencePilot software. The CCM3 KO population depicted here is compound heterozygous for the variants c.88_94del; p.(Phe30Serfs*2) and c.90dup; p.(Asn31*) [reference sequence: NM_007217.4].

Additional file 4. Lists and gene ontology analyses of commonly and exclusively deregulated genes in CCM3 KO and WT iECs, as shown in Figures 6C and 7A.

Additional file 5. Treatment of CCM3 KO and WT iEC co-cultures with semaxinib and different concentrations of U0126. The proportion of CCM3 KO cells (A) and total cell count (B) each normalized to the semaxinib-only treated condition and presented as means and SD (n = 3) are shown. Statistical analysis was performed using one-way ANOVA followed by Dunnett's multiple comparisons test, comparing each treatment to the semaxinib-only treated condition (first column) (* = Padj < 0.05, ** = Padj < 0.01, *** = Padj < 0.001).

Additional file 6. Table on VEGF signaling blockage in CCM studies.

## Data Availability

All relevant data generated or analyzed during this study are included in this published article and its supplementary information files. RNA sequencing data can be accessed through the Gene Expression Omnibus (GEO) database (record number: GSE310745).

## Abbreviations

2D: Two-dimensional
ACF: Animal component-free
AhR: Aryl hydrocarbon receptor
BSA: Bovine serum albumin
CCM: Cerebral cavernous malformation
DEGs: Differentially expressed genes
DMSO: Dimethyl sulfoxide
EC: Endothelial cell
ECM: Extracellular matrix
EDTA: Ethylenediaminetetraacetic acid
EGFP: Enhanced green fluorescent protein
FACS: Fluorescence-activated cell sorting
FGF-2: Fibroblast growth factor 2
HTS: High-throughput screening
iEC: iPSC-derived endothelial cell
iPSC: Induced pluripotent stem cell
KO: Knockout
PBS: Phosphate-buffered saline
PCR: Polymerase chain reaction
PFA: Paraformaldehyde
RFP: Red fluorescent protein
RNAi: RNA interference
RNA-seq: RNA sequencing
ROCK: Rho-associated protein kinase
SD: Standard deviation
VEGFA: Vascular endothelial growth factor A
VEGFR2: Vascular endothelial growth factor receptor 2
WT: Wild-type
