# Supplementary figures and images for: Tumor-like proliferation of CCM3 knockout endothelial cells: insights from semaxinib treatment and transcriptome profiling of co-cultures
Source: Acta Neuropathol Commun. 2026 Apr 20;14:103. doi: 10.1186/s40478-026-02283-1 (PMC13126972; doi:10.1186/s40478-026-02283-1)

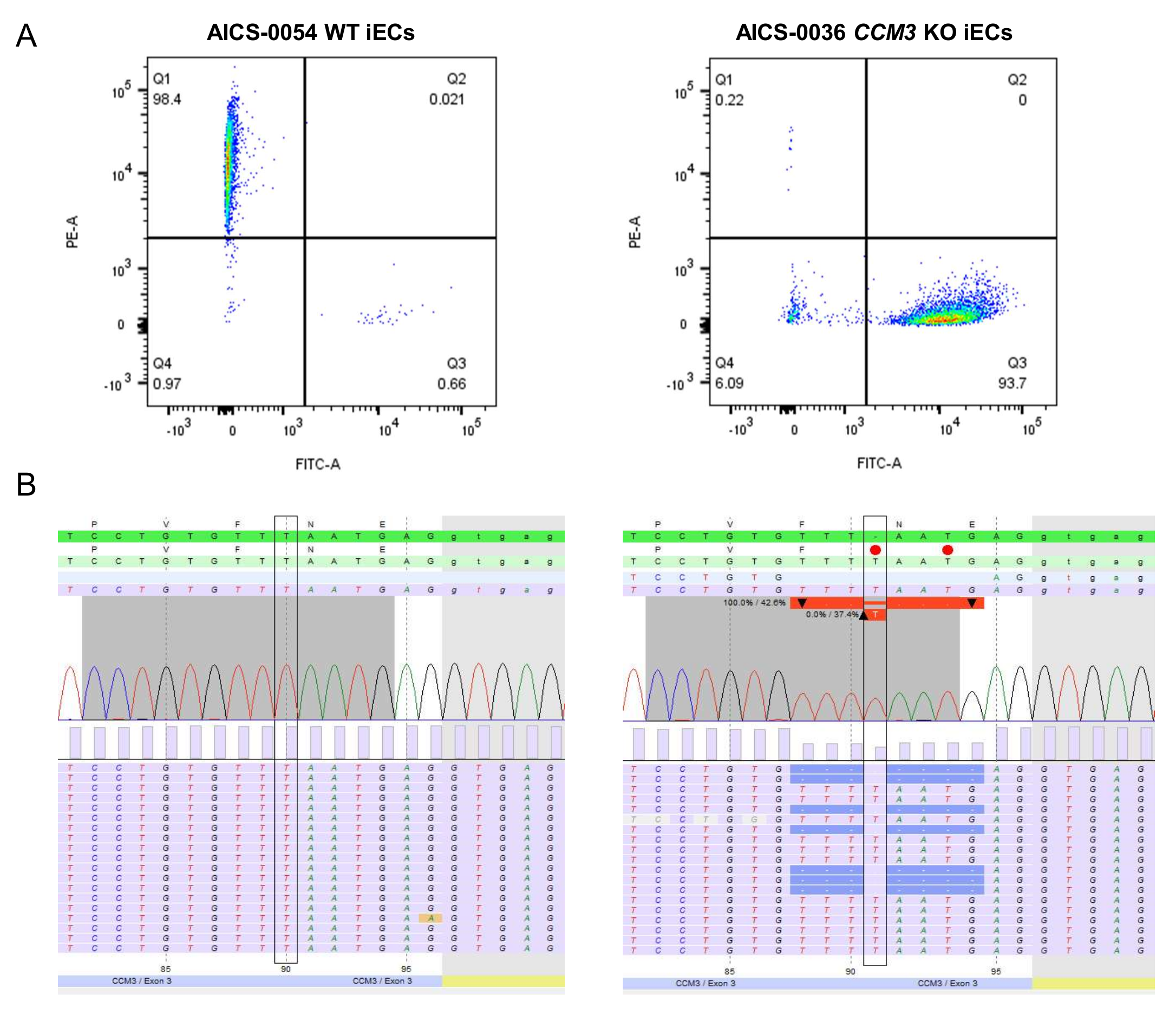

Supplement: Supplementary file 3 — Additional file 3. Confirmation of the high purity of sorted cell populations after FACS of WT and CCM3 KO iEC co-cultures for RNA-seq analysis. Exemplary depiction of post-sorting (A) and next-generation sequencing (NGS) data (B) of the sorted cell populations. The NGS data is shown as excerpts from the SequencePilot software. The CCM3 KO population depicted here is compound heterozygous for the variants c.88_94del; p.(Phe30Serfs*2) and c.90dup; p.(Asn31*) [reference sequence: NM_007217.4]. [file 40478_2026_2283_MOESM3_ESM.tiff]

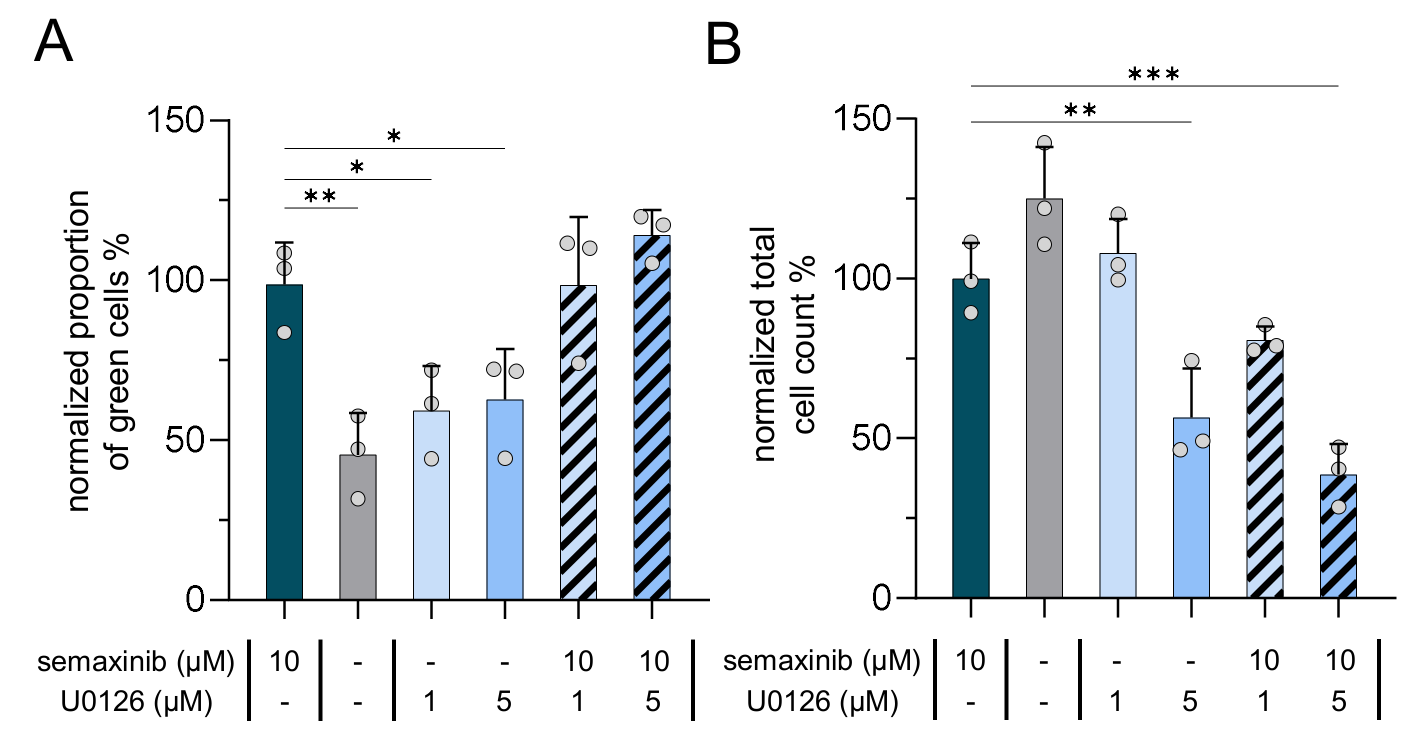

Supplement: Supplementary file 5 — Additional file 5. Treatment of CCM3 KO and WT iEC co-cultures with semaxinib and different concentrations of U0126. The proportion of CCM3 KO cells (A) and total cell count (B) each normalized to the semaxinib-only treated condition and presented as means and SD (n = 3) are shown. Statistical analysis was performed using one-way ANOVA followed by Dunnett's multiple comparisons test, comparing each treatment to the semaxinib-only treated condition (first column) (* = Padj < 0.05, ** = Padj < 0.01, *** = Padj < 0.001). [file 40478_2026_2283_MOESM5_ESM.tiff]
