# Supplementary material for: Tumor-like proliferation of CCM3 knockout endothelial cells: insights from semaxinib treatment and transcriptome profiling of co-cultures
Source: Acta Neuropathol Commun. 2026 Apr 20;14:103. doi: 10.1186/s40478-026-02283-1 (PMC13126972; doi:10.1186/s40478-026-02283-1)
Supplement: Supplementary file 6 — Additional file 6. Table on VEGF signaling blockage in CCM studies. [file 40478_2026_2283_MOESM6_ESM.docx]

**Additional file 6: VEGF signaling blockage in CCM studies.**

| **Substance** | **Function** | **Model** | **Outcome** | **Reference** |
| --- | --- | --- | --- | --- |
| VEGFR2/Fc | chimera protein, binds VEGF with high affinity, potent VEGF antagonist | HPAEC, BAEC  (*CCM1* siRNA knockdown) | - reduced CCM1 depletion-dependent VEGFR2 phosphorylation  - blocked stress fiber formation, reduced permeability and migration | DiStefano et al. 2014 |
| SU5416  (semaxinib) | potent and selective inhibitor of VEGFR | mouse (*Ccm1*^+/−^) | - reversed increase in permeability |  |
| SU5416  (semaxinib) | potent and selective inhibitor of VEGFR | mouse (*Ccm1*^ieKO^) | - reversed increased VEGFR2 phosphorylation  - reduced lesion number, but not lesion size  - blocked vascular permeability, reduced lesion haemorrhage | DiStefano and Glading 2020 |
| sorafenib | multikinase inhibitor (including VEGFR) | HUVEC  (*CCM1* siRNA knockdown) | - blocked VEGF-induced sprouting after *CCM1* silencing  - ameliorated excessive vasculature of CCM1-depleted HUVEC grafts in mice | Wüstehube et al. 2010 |

HPAEC: Human Pulmonary Artery Endothelial Cells, BAEC: Bovine Aorta Endothelial Cells

**References:**

DiStefano PV, Kuebel JM, Sarelius IH, Glading AJ (2014) KRIT1 protein depletion modifies endothelial cell behavior via increased vascular endothelial growth factor (VEGF) signaling. J Biol Chem 289:33054-33065. https://doi.org/10.1074/jbc.M114.582304

DiStefano PV, Glading AJ (2020) VEGF signalling enhances lesion burden in KRIT1 deficient mice. J Cell Mol Med 24:632-639. https://doi.org/10.1111/jcmm.14773

Wüstehube J, Bartol A, Liebler SS, Brütsch R, Zhu Y, Felbor U *et al* (2010) Cerebral cavernous malformation protein CCM1 inhibits sprouting angiogenesis by activating DELTA-NOTCH signaling. Proc Natl Acad Sci U S A 107:12640-12645. https://doi.org/10.1073/pnas.1000132107
